# Antibiofilm and Anti-Quorum Sensing Potential of Cycloartane-Type Triterpene Acids from Cameroonian Grassland Propolis: Phenolic Profile and Antioxidant Activity of Crude Extract

**DOI:** 10.3390/molecules27154872

**Published:** 2022-07-29

**Authors:** Alfred Ngenge Tamfu, Ozgur Ceylan, Geta Cârâc, Emmanuel Talla, Rodica Mihaela Dinica

**Affiliations:** 1 School of Chemical Engineering and Mineral Industries, University of Ngaoundere, Ngaoundere 454, Cameroon; tallae2000@yahoo.fr; 2 Food Quality Control and Analysis Program, Ula Ali Kocman Vocational School, Mugla Sitki Kocman University, Mugla 48147, Turkey; ozgurceylan@mu.edu.tr; 3 Department of Chemistry, Faculty of Sciences and Environment, Physics and Environment, Dunarea de Jos University, Galati, 47 Domneasca Str., 800008 Galati, Romania; getac@ugal.ro

**Keywords:** propolis, phenolic profile, antioxidant activity, cycloartane-type triterpene acids, antimicrobial, antibiofilm, anti-quorum sensing

## Abstract

Propolis is very popular for its beneficial health properties, such as antimicrobial activity and antioxidant effects. It is one of the most long-serving traditional medicines to mankind due to its interesting chemical diversity and therapeutic properties. The detailed chemical information of propolis samples is very necessary to guarantee its safety and for it to be accepted into health care systems. The phenolic profile of the hydroethanolic extract was determined using HPLC-DAD, and the antioxidant was evaluated using five complementary methods. Triterpenoids were isolated using column chromatography and characterized using ^1^H NMR and ^13^C NMR. The effects of the extract and the isolated compounds on quorum sensing mediated processes and biofilm formation in bacteria were evaluated. Protocatechic acid (40.76 ± 0.82 µg/g), 4-hydroxybenzoic acid (24.04 ± 0.21 µg/g), vanillic acid (29.90 ± 1.05 µg/g), quercetin (43.53 ± 1.10 µg/g), and luteolin (4.44 ± 0.48 µg/g) were identified and quantified. The extract showed good antioxidant activity in the DPPH^•^, ABTS^•+^, CUPRAC, and metal chelating assays, and this antioxidant effect was confirmed by cyclic voltammetry. 27-Hydroxymangiferonic acid (1), Ambolic acid (2), and Mangiferonic acid (3) were isolated from anti-quorum sensing activity at MIC, and it was indicated that the most active sample was the extract with inhibition diameter zone of 18.0 ± 1.0 mm, while compounds **1**, **2**, and **3** had inhibition zones of 12.0 ± 0.5 mm, 9.0 ± 1.0 mm, and 12.3 ± 1.0 mm, respectively. The samples inhibited the *P. aeruginosa* PA01 swarming motility at the three tested concentrations (50, 75, and 100 μg/mL) in a dose-dependent manner. The propolis extract was able to inhibit biofilm formation by *S. aureus*, *E. coli*, *P. aeruginosa*, *C. albicans*, and *C. tropicalis* at MIC concentration. Compound **1** proved biofilm inhibition on *S. aureus*, *L. monocytogenes*, *E. faecalis*, *E. coli*, and *C. tropicalis* at MIC and MIC/2; compound **2** inhibited the formation of biofilm at MIC on *S. aureus*, *E. faecalis*, *E. coli*, *S. typhi*, *C. albicans*, and *C. tropicalis*; and compound **3** inhibited biofilm formation on *E. faecalis*, *E. coli*, *C. albicans*, and *C. tropicalis* and further biofilm inhibition on *E. coli* at MIC/4 and MIC/8. The studied propolis sample showed important amounts of cycloartane-type triterpene acids, and this indicates that there can be significant intra-regional variation probably due to specific flora within the vicinity. The results indicate that propolis and its compounds can reduce virulence factors of pathogenic bacteria.

## 1. Introduction

It is reported that about 400 million years of life loss that continuously occur annually in the world are caused by pathogenic microbes, and as the population of the world increases with more interconnectivity the infectious disease risk is aggravated and creates a human health burden worldwide [1]. Foodborne disease (infectious disease that results from food consumption) can threaten the life of the affected person and mostly individuals with weak immune systems, pregnant women, older people, and very young children are at higher risk [2]. Many parasitic viruses, bacteria, and fungi are responsible for foodborne disease, and the inhibition of these microbes is a suitable control method. Resistance often arises when these parasites are no longer susceptible to antibiotics used against them, thereby constituting an emerging and life-threatening global health problem that requires an imperative and urgent need for action to avert this developing global health care crisis [3]. The uncontrolled use of antibiotics results in the development of multidrug-resistant strain microorganisms living within protective shield known as biofilms, due to gene expression modulation of microorganism virulence factors caused by selective pressure exerted on their survivability instead of killing them [4]. Since microbial biofilm formation is a serious medical threat, it is urgent to find new bioactive principles such as compounds and crude medicinal extracts from natural products such as plants to treat infectious diseases caused by multidrug-resistant bacteria [5]. Microbial biofilms consist of communities of microbial cells densely packed on biotic and abiotic surfaces within a polymeric matrix, which needs very high doses of antibiotics (10–1000 times) to kill the bacterial cells and are responsible for persistent chronic infections [6]. Biofilm protects bacteria cells against several adverse physicochemical conditions such as heavy metals, ultraviolet light, acidity, modulation in hydration or salinity, and phagocytosis [7,8]. Quorum sensing (QS) depends on the density of bacterial cells, and it is a signaling network that promotes bacterial cell–cell communication. It regulates virulence factors including violacein pigment, motilities, and biofilm development, which foster the establishment of chronic infections; hence, the disruption of QS is an effective strategy to control and eliminate various virulence factors [9]. Additionally, many endogenous factors including the effects of ROS (superoxide anion radical, nitric oxide radical, hydroxyl radical, hydrogen peroxide, hypochlorite radical, singlet oxygen) as well as exogenous causes such as ionizing radiation, smoking, organic solvents, pollution, and pesticides result into oxidative stress because they can attack biomolecules such as proteins, nucleic acids and enzymes leading to deformation of their structures and functions [10]. It is therefore necessary to seek antioxidant substances that can help to stop the devastating effects resulting from oxidative damage.

Recently, natural products, besides their uses as traditional therapeutics, find increasing applications due to their biological and ecological functions in regulating interactions between microorganisms. This is so because plants defend themselves from pathogens not by immunity but by biochemical systems of defense and are able to produce anti-QS compounds to overcome QS-dependent microbes [11]. Propolis is a plant-derived natural product resulting from resins from buds, exudates, and other parts of plants, mixed with salivary enzymes and beeswax, and it possesses wide spectrum of bioactivities due to its complex and variable chemical composition [11,12]. The chemical composition as well as bioactivities of propolis samples vary from one geographical location to another and also depends on the season of collection and bee species [13]. Propolis, also called bee glue, is sticky and made up of resins together with waxes, volatile oils, polyphenols, polysaccharides, and diverse natural compounds that confer numerous medicinal properties such as antiulcer, antibacterial, anti-inflammatory, antioxidant, anti-angiogenic, and antiviral activities [14]. Propolis is the chemical weapon of bees within the hive used to deter bacteria and other microbes and to embalm and mummify dead invaders, and man has learned to use it mainly as an antibiotic and as a powerful antioxidant capable of modulating the action of reactive oxygen species within the human body [15,16]. The antimicrobial and antioxidant properties enable propolis to find applications in the food industry as it can delay lipid oxidation and increase the shelf life of food stuffs [15]. Over 300 compounds have been isolated from propolis from different regions of the world but there is no clear-cut distinction of propolis from different regions, but the major chemical compounds can be classified in two main groups as those from temperate propolis and those from tropical propolis. Compounds isolated from propolis from temperate regions are mostly flavones, flavonols, flavanones, flavanonols, chalcones, aurones, pterocarpans, lignans, phenolic acids, and their esters, etc. Compounds isolated from propolis from tropical and subtropical regions are mostly terpenoids, steroids, and xanthone [17]. As expected, propolis samples from tropical areas are scientifically proven to be rich in variable chemical structures, mostly terpenoids, lignans, flavonoids, and prenylated organic acid derivatives [18]. Previous studies on Cameroonian propolis have described the presence of pentacyclic triterpenoids as major constituents; several of them have been isolated and characterized, and they have demonstrated interesting antimicrobial activities [13,14,19,20,21,22,23].

Cameroon is found within tropical Africa, and there has been an increase in beekeepers, from over 20,000 beekeepers in 2009, who market important amounts of honey, waxes, and propolis [24]. Important amounts of propolis are produced in Cameroon, and this product is gaining attention in traditional medicine as well as scientific research for biological properties and chemical composition. The aim of this study was to prepare hydroethanolic (70%) extract of propolis, to determine the phenolic content using High-Performance Liquid Chromatography with Diode Array Detector (HPLC-DAD), and to evaluate the antioxidant activity as well as highlighting redox processes by cyclic voltammetry. Secondly, this work aimed at isolating chemical constituents from the extract and evaluating their inhibitory effects on microbial virulence factors such as biofilm formation and quorum sensing.

## 2. Results

### 2.1. HPLC-DAD Phenolic Profile and NMR Data of Isolated Compounds

**Figure 1:**
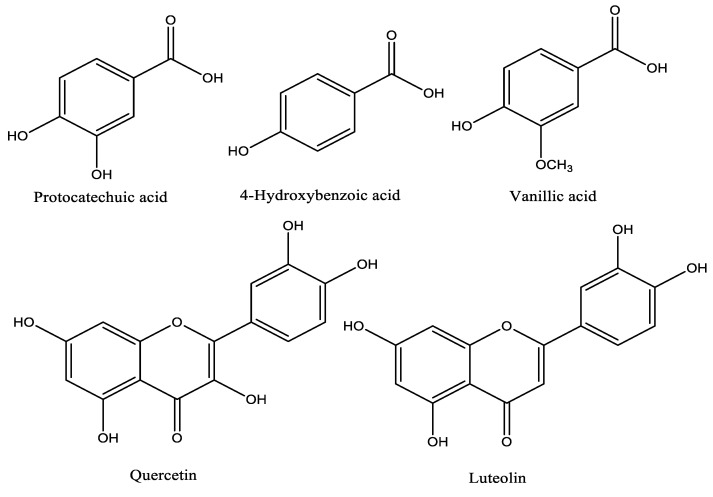
Phenolic compounds detected in the propolis extracts by HPLC-DAD.

The importance of phenolic compounds in food and human health has been proven in many studies and for this reason, the extraction, characterization, and evaluation of bioactivity of phenolic compounds have attracted much interest from researchers. In this study, the phenolic profile of propolis sample extract determined using HPLC-DAD with internal standard phenolics is given on Table 1. Five phenolic compounds—protocatechic acid (40.76 ± 0.82 µg/g), 4-hydroxybenzoic acid (24.04 ± 0.21 µg/g), vanillic acid (29.90 ± 1.05 µg/g), quercetin (43.53 ± 1.10 µg/g), and luteolin (4.44 ± 0.48 µg/g)—were identified and quantified while catechin, 6,7-dihydroxycoumarin, vanillin, *p*-coumaric acid, and trans cinnamic acid were detected at trace levels. The structures of the identified compounds are given in Figure 1.

**Figure 2:**
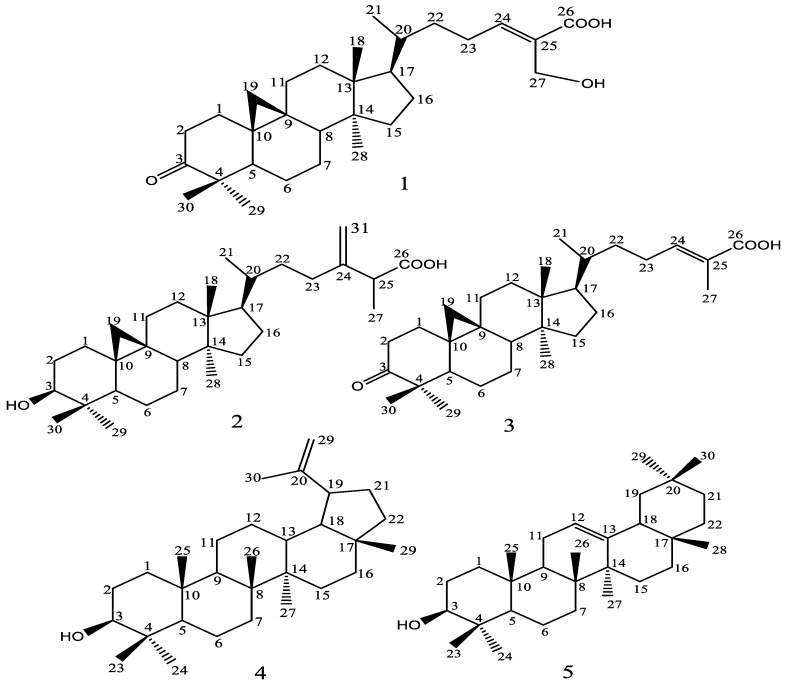
Structures of chemical compounds isolated from propolis sample; 27-Hydroxymangiferonic acid (1), Ambolic acid (2), Mangiferonic acid (3), Lupeol (4), β-amyrin (5).

Column chromatographic purification of the extract afforded five compounds which were characterized as 27-Hydroxymangiferonic acid (1), Ambolic acid (2), Mangiferonic acid (3), lupeol (4), and β-amyrin (5). It is worthy to note that the isolated compounds are all pentacyclic triterpenoids, and terpene derivatives are known to be characteristic constituents of tropical propolis samples, occurring in high amounts and easy to isolate. The NMR data of the isolated compounds are given on Table 2 while their structures are given in Figure 2. The compounds isolated in good amounts, particularly 27-Hydroxymangiferonic acid (1), Ambolic acid (2), and Mangiferonic acid (3) were also tested for their effects on microbial virulence factors.

### 2.2. Antioxidant Activity

It is very necessary to seek for antioxidant substances that can stop or delay the adverse health effects of reactive oxygen species (ROS) and other oxidants in the body. Natural substances with antioxidant properties are of major importance in the prevention of diseases. The antioxidant activity of the propolis extract was evaluated using four different models, which are DPPH^•^, ABTS^•+^, CUPRAC, and metal chelating assays since antioxidant capacity cannot be concluded based on a single method. The results of the antioxidant assay are given in Table 3. In the DPPH^•^ assay, the propolis extract had an IC_50_ value of 14.90 ± 1.10 µg/mL, and this is good activity as it was close to the activity exhibited by the standards α-tocopherol (12.26 ± 0.07 µg/mL), BHT (25.37 ± 0.47 µg/mL), and quercetin (2.07 ± 0.10 µg/mL). On the radical cation ABTS^•+^, the IC_50_ value of the propolis extract was 10.86 ± 0.91 µg/mL compared to α-tocopherol (4.31 ± 0.10 µg/mL), BHT (4.10 ± 0.06 µg/mL), and quercetin (1.18 ± 0.03 µg/mL). In the CUPRAC assay, the A_0.50_ of the propolis extract was 6.50 ± 0.25 µg/mL indicating good activity when compared to α-tocopherol (10.20 ± 0.01 µg/mL) and BHT (3.80 ± 0.00 µg/mL). The propolis extract was also able to exhibit metal chelation with a percentage inhibition of 46.21 ± 0.71% when compared to quercetin (35.91 ± 0.82%) and EDTA (85.40 ± 0.10%). The propolis extract exhibited higher activity than the standard α-tocopherol in the DPPH^•^ and CUPRAC assays, and EDTA in the metal chelating assay.

### 2.3. Violacein Inhibition and Anti-Quorum Sensing Activity

Chromobacterium produces the purple violacein pigment while growing, and this violacein pigment acts as an antioxidant protecting the bacterial membrane against oxidative stress through a quorum sensing mediated process. The bacterial strain *C. violaceum* CV12472 is usually employed in qualitative screening of inhibition of violacein production, which is revealed by the absence or reduction of the violet coloration. Prior to the violacein inhibition, the MIC values of the extract and compounds were determined and the violacien inhibition assay was carried out at MIC and sub-MIC concentrations. The MIC values were 0.5 mg/mL for the extract and mangiferonic acid and 1 mg/mL for 27-hydroxymangiferonic acid and ambolic acid on *C. violaceum* CV12472. All test samples exhibited excellent violacein inhibition with percentages of inhibition of 100% at MIC and MIC/2 concentrations. The most active samples in this assay were 27-hydroxymangiferonic acid and ambolic acid as they showed 100% inhibition right at MIC/4 concentration and were the only samples to inhibit violacein beyond MIC/8. 27-hydroxymangiferonic acid and ambolic acid had violacein inhibition percentages of 35.89 ± 0.64% and 24.90 ± 1.00% at MIC/16 and no sample was able to inhibit violacein at MIC/32.

The mutant strain CV026 does not produce violacein while growing except when an acylhomoserine lactone (AHL) hormone is supplied to it externally, and this bacterium can be used to determine the disruption of quorum sensing by determining the quorum sensing inhibition zones. The MIC values of the extract and compounds on *C. violaceum* CV026 were determined prior to determination of quorum sensing inhibition zones. The MIC values were 0.25 mg/mL for the extract, 1 mg/mL for ambolic acid, and 0.5 mg/mL for 27-hydroxymangiferonic acid and mangiferonic acid. All samples showed inhibition of quorum sensing at MIC concentration. The most active sample was the extract with inhibition diameter zone of 18.0 ± 1.0 mm, while 27-hydroxymangiferonic acid, ambolic acid, and mangiferonic acid had inhibition zones of 12.0 ± 0.5 mm, 9.0 ± 1.0 mm, and 12.3 ± 1.0 mm, respectively. Only the extract inhibited quorum sensing beyond MIC precisely at MIC/2 with inhibition zone of 14.5 ± 0.5 mm. The results for violacein inhibition and anti-QS are given on Table 4 and Table 5, respectively.

### 2.4. Swarming Motility Inhibition

Swarming motility of microorganisms is implicated in QS-mediated biofilm formation, and it is most especially important in flagellated bacteria such as *P. aeruginosa* PA01, the strain that is considered as a model for this assay. The inhibition of swarming movement of the samples was evaluated at three concentrations of 50, 75, and 100 μg/mL and results are presented on Table 6. The samples inhibited the *P. aeruginosa* PA01 swarming motility at the three tested concentrations (50, 75, and 100 μg/mL) in a dose-dependent manner and all samples showed activity at 100 and 75 µg/mL, but only ambolic acid and mangiferonic acid showed inhibitions at 50 µg/mL. Ambolic acid was the most active sample with percentage inhibitions of 58.72 ± 1.05%, 31.80 ± 0.91%, and 6.68 ± 0.70% at 100, 75, and 50 μg/mL concentrations, respectively.

### 2.5. Antimicrobial and Antibiofilm Activities

An attempt to overcome the issue of microbial resistance is the search for new antimicrobial substances from natural sources, and propolis is a good potent source. When bacteria are able to avoid being inhibited by antibiotics, a common explanation will be the formation of biofilms, which are complex protective matrices within which communities of sessile bacteria can survive even under harsh conditions such as starvation, host defenses, and antibiotics. Biofilms can get attached on living and nonliving surfaces, and even when planktonic bacteria die, those within biofilms will persist; thus, biofilm formation is a mode of resistance to antibiotics. Prior to the evaluation of biofilm inhibition, the antimicrobial activity of the extract and compounds was evaluated against three gram-positive bacteria (*S. aureus* ATCC 25923, *L. monocytogenes* ATCC 7644, and *E. faecalis* ATCC 29212), three gram-negative bacteria (*E. coli* ATCC 25922, *P. aeruginosa* ATCC 27853, and *S. typhi* ATCC 14028) and two yeast (*C. albicans* ATCC 10239 and *C. tropicalis* ATCC 13803) and the results are presented in Table 7. The maximum test concentration was 1 mg/mL, and the MIC values ranged from 0.25 to 1 mg/mL in case of activity while on some bacteria and for some compounds, the MIC values were not within the tested concentrations, that is >1 mg/mL. The most susceptible microorganism was *S. aureus* on which the extract and mangiferonic acid had MIC values of 0.25 mg/mL and 27-hydroxymangiferonic acid and ambolic acid had MIC values of 0.5 mg/mL. *C. albicans*, *C. tropicalis,* and *E. faecalis* were susceptible to the extract and compounds as their MIC values were all within tested concentrations. The percentages of biofilm inhibition assayed at MIC and sub-MIC concentrations are reported in Table 8. For the propolis extract, it was able to inhibit biofilm for *S. aureus*, *E. coli*, *P. aeruginosa*, *C. albicans,* and *C. tropicalis* at MIC concentration. The highest inhibition was exhibited on *E. coli* and was 38.5 ± 1.2% and 20.5 ± 0.3% at MIC and MIC/2, respectively. The propolis extract also inhibited biofilm formation for *S. aureus* MIC (18.3 ± 0.8%) and MIC/2 (5.1 ± 0.2%). 27-Hydroxymangiferonic acid showed biofilm inhibition on *S. aureus, L. monocytogenes, E. faecalis, E. coli,* and *C. tropicalis* at MIC and MIC/2. *C. albicans* biofilm inhibition occurred only at MIC while *S. aureus, E. coli,* and *C. tropicalis* biofilm formation was inhibited at MIC/4 and for *E. coli*, further inhibition was observed at MIC/8. Good inhibitions by 27-hydroxymangiferonic acid were observed on *S. aureus* at MIC (40.5 ± 0.9%), MIC/2 (25.5 ± 0.2%), and MIC/4 (8.8 ± 0.3%), on *E. coli* at MIC (38.5 ± 1.1%), MIC/2 (25.8 ± 0.8%), MIC/4 (15.6 ± 0.6%), and MIC/8 (8.3 ± 0.4%), and on *C. tropicalis* at MIC (40.1 ± 1.0%), MIC/2 (21.8 ± 0.4%), and MIC/4 (7.9 ± 0.1%). Ambolic acid inhibited the formation of biofilm at MIC on *S. aureus, E. faecalis, E. coli, S. typhi, C. albicans,* and *C. tropicalis*. At MIC/2, *S. aureus, E. coli,* and *C. tropicalis* biofilms were inhibited, and it was only in *E. coli* that biofilms were further inhibited at MIC/4 and MIC/8. Good activity of ambolic acid was observed on *C. tropicalis* at MIC (39.8 ± 1.0%), MIC/2 (26.0 ± 0.9%), and MIC/4 (10.2 ± 0.4%) and on *E. coli* at MIC (32.5 ± 0.5%), MIC/2 (21.8 ± 0.7%), MIC/4 (11.9 ± 0.2%), and MIC/8 (3.3 ± 0.1%). Mangiferonic acid exhibited inhibition at MIC for *S. aureus, L. monocytogenes, E. faecalis, E. coli, S. typhi, C. albicans,* and *C. tropicalis*. At MIC/2, mangiferonic acid inhibited biofilm formation on *E. faecalis, E. coli, C. albicans,* and *C. tropicalis* and further biofilm inhibition on *E. coli* at MIC/4 and MIC/8. Good activity of this compound was observed on *E. faecalis* at MIC (32.4 ± 0.9%) and MIC/2 (12.6 ± 0.2%), on *C. albicans* at MIC (44.5 ± 0.4%) and MIC/2 (26.1 ± 0.5%), and on *E. coli* at MIC (44.5 ± 1.0%), MIC/2 (28.9 ± 0.8%), MIC/4 (12.1 ± 0.2%), and MIC/8 (5.2 ± 0.1%).

## 3. Discussion

The solvent used for obtaining the crude extract was a hydroethanol solution (70:30; ethanol:water; *v/v*) due to its non-toxicity and suitability in the extraction of propolis as it limits the amounts of sticky substances, oils, and lipids in the crude extracts. The phenolic profile was analyzed against 26 standard phenolics by HPLC-DAD and showed low quantities of phenolics, as will be expected from tropical propolis samples. The ethanol/water mixture (70%) has optimizing influence on phenolic profile and antimicrobial and antioxidant activities [25,26,27]. Propolis has been shown to possess phenolics such as p-coumaric acid, caffeic acid, luteolin, ellagic acid, vitexin, quercetin, rutin, apigenin, prenylated benzophenones, and also many terpenoids which are responsible for the most attractive activity of propolis, the antibacterial activity [28]. The difference in composition can be explained by the climatic and floral differences. Though in small amounts, phenolic compounds can play an important role in the bioactivities of propolis samples. Phenolic compounds have antioxidative properties and can be used in food to prevent or delay oxidation of fats and oils, and there is growing interest in these natural antioxidants as they can be used as food preservatives and for other human health benefits [29].

The use of synthetic antioxidants is on a decline because compounds such as butylated hydroxytoluene (BHT) and butylated hydroxyanisole (BHA) may have adverse effects and as a result, there is a need to identify alternative natural sources of food antioxidants [30]. Propolis extracts are consumed by people from different parts of the world in the form of alternative medicine, food complements and nutraceuticals since they have antioxidant properties and can contribute protect living tissues from cellular damage caused by oxidative agents and free radicals [31].

The notable phenolic compounds detected in this propolis sample are protocatechic acid, 4-hydroxybenzoic acid, vanillic acid, quercetin, and luteolin, which are known propolis phenolic constituents previously identified in propolis and contribute to the antioxidant capacity of propolis [32,33]. The propolis extract in this study exhibited good antioxidant capacity in the DPPH^•^, ABTS^•+^, CUPRAC, and metal chelating assays. Electron donation mechanism is the mode of action of classical antioxidant substances [34]. Single-electron transfer methods are based primarily on deprotonation and the ionization potential of the reactive functional group and are hence pH-dependent [27,35]. The tested propolis sample has been shown to contain phenolic antioxidants that have multiple functional groups, and therefore their antioxidant capacities could not be suitably determined through a simple method because in this study, a combination of spectrophotometric techniques—which are DPPH^•^, ABTS^•+^, CUPRAC, and metal chelating assays—were used. The results from these various methods give a proper indication of the antioxidant property of the propolis sample. Additionally, the cyclic voltammetry, an electrochemical technique, was tested on the propolis sample. The quantification of phenolic compounds in vegetables, foods, and plants is estimated through electrochemical methods, and the over-all amount of each type of polyphenol is also estimated, which is very useful in pharmaceutical and food industries. Cyclic voltammetry based on registration of the anodic current indicates that a compound donates electrons, which is useful for phenolic content and antioxidant capacity [36,37].

In this way, the cyclic voltammetry (CV) could provide useful information about the redox process for the propolis extract. The peak of anodic current, I_p,a_, towards larger values (until 7.22 μA) at E applied of ±2 V/Ag/AgCl_sat_. was registered when it was compared with potential applied of E of ±1 V/Ag/AgCl_sat_. (an I_p,a_ of 1.98 μA) (not shown graphs). When there is an increase of scan rate from 20 mV·s^−1^ to 50 mV·s^−1^ and finally 100 mV·s^−1^, respectively, in voltammograms a slow increase of the peak oxidation current was registered, explained by stability of the compounds involved in the exchange of electrons (not shown graphs). The result at different potentials opens up new possibilities for the study of the properties or the kinetics of chemical systems involved in propolis anti-oxidative activity.

This could be the response from different antioxidants compounds, probably the phenolic compounds that have different oxidation potentials. The oxidation of hydroxyl groups of the molecules, probably the phenolic compounds detected in the propolis extract by HPLC-DAD (protocatechuic acid, 4-hydroxybenzoic acid, vanillic acid, quercetin, and luteolin) could constitute a main component that accounts for these results. The oxidation of the phenolic compounds, represented by the top scan, generates a positive anodic current while the reverse scan produces a negative cathodic current, indicating that the oxidized species is reversibly reduced to its original form [38]. Cyclic voltammetry is a good indicator for checking the oxidation potential of extracts or phenolic compounds and therefore could serve as a suitable alternative to spectrophotometric methods in the evaluation the antioxidant activity [38]. The results at different potentials open up new possibilities for the study of the properties or the kinetics of chemical systems involved in propolis anti-oxidative activity. 

For the reason that bees use propolis mainly as a chemical weapon against intruders and for keeping the beehive disinfected, the antimicrobial activity of propolis will be the most important hypothesis for scientific research. Propolis has shown antimicrobial activity as crude extracts and also in synergy with antimicrobial drugs, and this association with commercially available drugs shows interesting leads for the development of new products for pharmaceutical industry [39]. The studied propolis sample showed high amounts of cycloartane-type triterpene acids, and Cameroonian propolis samples have shown similar constituents but with smaller amounts This shows that despite similarities in phytoconstituents of propolis within a geographical zone, there can still be important and significant intra-regional variation probably due to specific flora within the vicinity. The results obtained here show that propolis extract and its constituents are capable of inhibiting bacterial growth although different microorganisms showed different susceptibilities to each of the tested samples, meaning that one sample cannot inhibit all bacteria to the same extent. Several factors, such as the nature of microorganisms, the inoculum or cell concentration as well as mode of action, influence the antimicrobial property of propolis, and it is well-established that propolis is capable of inhibiting the growth of a wide range of microorganisms, either gram-positive, gram-negative, or yeasts [40]. In the results, it can generally be observed that in most of the samples, activity was higher in gram-positive bacteria (*S. aureus, L. monocytogenes,* and *E. faecalis*) than on the gram-negative bacteria (*E. coli, P. aeruginosa,* and *S. typhi*), but there was an exception between only the crude extract where *S. typhi* (gram-positive) was more susceptible to the propolis extract than *E. faecalis* (gram-positive). This corroborates with the fact that gram-positive bacteria are generally more susceptible to propolis and its constituents and therefore can only be inhibited at higher doses. This may be due to that fact the presence of efflux pumps that are able to prevent propolis and its constituents from entering the bacterial cells (intracellular entry), creating greater resistance of gram-negative bacteria to propolis than the gram-positive [41]. Since propolis has constituents that are mainly derived from vegetal plant resins, which are secreted by plants to combat and protect them mostly from gram-positive pathogens, propolis is likely to have low activity and weak response on gram-negative bacteria [42]. Propolis and its constituent compounds act as natural antimicrobials over a broad spectrum of different bacteria capable of reducing the antimicrobial resistance of bacteria, and this activity can vary according to the chemical classes of the propolis constituents, which is dependent on regional and seasonal factors [15]. This antimicrobial activity can be mainly attributed to the phenolic compounds detected in the extract as well the triterpenoid compounds isolated and tested. Propolis contains many compounds, including phenolic compounds, flavonoids, isoflavonoids, prenylated benzophenones, caffeic acid, ellagic acid, p-coumaric acid, apigenin, vitexin, luteolin, quercetin, rutin, and triterpenoids, and these compounds confer mainly antibacterial activity on propolis [28]. The propolis extract and triterpenoid compounds in this study demonstrated antimicrobial activity and inhibited biofilm development on certain microbial strains.

Although many studies report antimicrobial activity of propolis and its constituents, reports on the antibiofilm and quorum sensing potential of propolis are still scarce. Bacteria, when growing, may organize themselves into planktonic and sessile (biofilm) communities, and the difference is that planktonic bacteria are inhibited by antibiotics while sessile bacteria remain safe since biofilms protect them from most conventional antibiotics against which they become resistant, subsequently becoming able to evade the host defenses, causing chronic and untreatable infections [31]. Therefore, biofilms are very dangerous and are responsible for virulence of microbial infections and development of resistance towards conventional antimicrobials. Therefore, the capacity of the propolis extract and the isolated compounds to reduce biofilm formation through inhibition as shown in this study is a good indication of their possible application in the elimination of resistance and virulence during infections, which constitutes a major health burden and threat. Biofilms can cause chronic infection of the lungs that is a severe pathological condition associated with cystic fibrosis in patients and mostly due to drug-resistant biofilms formed around the bronchial mucus where there is equally high concentration of reactive oxygen species resulting from neutrophil activity [31]. Propolis extract was previously shown to significantly reduce total biomass and the number of viable bacterial cells, and it was shown that the cells were damaged and disorganized both in the planktonic cells and sessile biofilm cells caused by treatment with propolis [43]. The good antimicrobial activity and moderate antibiofilm activity reported here is contrary to the good antibiofilm activity of propolis extracts reported against gram-positive and gram-negative bacteria, which effectively reduce or eliminate biofilms and planktonic cell growth [31,44,45]. However, moderate to low susceptibility of biofilms to propolis extracts were reported by other researchers [46,47] and hence are in conformity with our results. The differences in the results can be explained by the variation in the propolis chemical compositions as well as the susceptibility of the different bacterial strains used in the different assays. As would be expected of tropical propolis samples, the studied Cameroonian propolis sample contained mostly triterpenes, and all the compounds isolated were pentacyclic triterpenoids. Despite the good antimicrobial activity of Cameroonian propolis and its constituents, antibiofilm and anti-quorum sensing activities of these constituents are not reported yet. It is crucial to find and evaluate new therapies that can treat bacterial biofilm formation and disrupt quorum sensing in bacteria so as to reduce the incidence and emergence of resistance strains. Triterpenes could find applications in this domain since some triterpenes and their derivatives have been shown to possess antimicrobial activities against planktonic and biofilm cells evaluated using the crystal violet method [48]. Biofilm matrices act as a barrier to antibiotics, preventing the penetration of antimicrobial agents, and the search for new molecules that can eliminate adhesion of bacterial cells and biofilms is growing tremendously [49]. Bacterial cells within biofilms can become 10 to 1000 times more resistant than their planktonic counterparts even if they are of the same strains [50]. Pentacyclic triterpenes possess antibacterial activity, and this could be because they cause changes in the structure and functioning of the bacterial cell membrane, morphology, gene expression, and processes such as adhesion and biofilm formation [51].

In this study, all the cycloartane-type triterpenic acids demonstrated good biofilm inhibition on *E. coli* and *C. tropicalis*. The antibiofilm activities were low on *L. monocytogenes*, *P. aeruginosa,* and *S. typhi*. The bacteria *E. coli* showed the highest biofilm inhibition susceptibility to the cycloartane-type triterpenic acids tested while the biofilms of *P. aeruginosa* were not disrupted by the cycloartane-type triterpenic acids. Previously, pentacyclic triterpenoid acids have been shown to exhibit antibiofilm and anti-quorum sensing activities on pathogenic bacteria [52,53,54]. This justifies the antibiofilm activity exhibited by the cycloartane-type triterpenoid acids tested in our study. It was suggested that triterpenes with more hydroxyl groups (more polar groups) are more hydrophilic in nature, and this makes it easier for them to penetrate inside the polymeric matrix of exopolysaccharides in bacterial biofilm and have an effect on the bacterial cells that are within the biofilm [51,54,55]. The polar group such as carboxyl, hydroxyl, and carbonyl groups present in these compounds could be responsible for this effect. The effects of natural products on bacterial biofilm result mostly from the inhibition of exopolymeric matrix formation and the suppression of cell adhesion, thereby disrupting the establishment of the extracellular matrix, reducing virulence factors, and blocking quorum sensing communications and growth of the biofilm [56]. Several natural compounds have shown antibiofilm and anti-quorum sensing activities, and they are promising antimicrobial substances since, in addition to their activities, the natural products extracts, essential oils, and phytochemicals have high efficacy as biofilm inhibitors and anti-quorum sensing agents with greater chemical stability, small molecular weights, and low toxicity to human health [9,52,57,58,59,60,61]. The results obtained in this study can be further supported by the fact that various bee products such as honey and propolis have been reported to possess good antimicrobial, antibiofilm, and anti-quorum sensing activities against pathogenic bacteria [59,62].

Quorum sensing involves a cell–cell communication process in bacteria through the secretion, detection, and response to extracellular signaling molecules, which enable the bacterial community to monitor information about the environment, population, and cell numbers and collectively alter their gene expression, helping them to act in synchrony [63]. This accounts for the virulence factors, biofilms, and severity during infections; therefore, it will be beneficial to inhibit and disrupt quorum sensing networks in pathogenic bacteria, especially with the use of natural medicinal plants and their compounds and essential oils [9,64,65]. The propolis sample and the isolated pentacyclic triterpenoids inhibited violacein production and quorum sensing in *C. violaceum* CV12472 and *C. violaceum* CV026, respectively, indicating that they can reduce bacterial cell-to-cell communication networks. Some propolis samples exhibited quorum sensing inhibitory (QSI) activity using the acyl-homoserine lactone-dependent *Chromobacterium violaceum* strain CV026, and one of the extracts showed a chemical profile with a high content of terpenoids [66]. Terpenoids and phenolic compounds contained in natural products are usually responsible for the anti-QS activity, and all these classes are contained in propolis and propolis samples containing various phenolic and terpenoid components that have been reported for anti-QS activity, violacein inhibition, and swarming/swimming motilities inhibition [67,68,69,70]. Bacterial motility is a quorum sensing mediated process that involves swarming and swimming movements, powered by flagella in bacteria such as *P. aeruginosa* PA01, which enables them to move to and attach themselves on surfaces before colonizing the surfaces and establishing biofilms [9,71,72]. Inhibiting swimming and swarming motilities therefore can greatly reduce microbial resistance and the incidence of surface colonization and biofilm formation on surfaces in general and most especially on medical devices and implants in particular, and this can eradicate community-acquired and healthcare-associated infections.

## 4. Materials and Methods

### 4.1. Propolis Collection and Extraction

The propolis was harvested from the grassland area usually referred to as ‘grassfield’, precisely from beehives in Babanki village (6°7′0″ N and 10°15′0″ E), in the north-west region of Cameroon in January 2018. In total, 100 g of the propolis were macerated in 1000 mL of hydro-ethanol solution (70%) with intermittent stirring for 48 h, after which the supernatant was decanted and filtered. The filtrate was then freeze-dried to yield 17 g of dry crude propolis extract that was stored at 4 °C prior to analyses.

### 4.2. Isolation and Characterization of Compounds

A portion of 5 g of the crude ethanol extract of propolis was dissolved in ethyl acetate, and silica gel was added to it to obtain a slurry. The slurry was then subjected to flash column chromatography (CC) over 60 g of silica gel with increasing gradient solvent polarity from CH_2_Cl_2_/AcOEt (0–100%) and collecting 50 mL volumes, followed by evaporation of rotavapor and TLC. The fractions were grouped on the basis of TLC profiles into 5 fractions (F1-F5). Fraction F2 (450.5 mg) was purified by CC over silica gel by elution with CH_2_Cl_2_/AcOEt (10–25%) to obtain 18 mg of the mixture of compound 4 (lupeol) and compound 5 (*β*-amyrin). Fraction F4 and F3 were combined (116.5 mg) and purified using CC on silica gel with an eluent of CH_2_Cl_2_/AcOEt (30%) to obtain 9 sub-fractions (f1-f9). Sub-fractions f1 to f5 crystallized on standing and were filtered out to give a total of 32 mg of compound 3 (mangiferonic acid) while sub-fraction f7 and f8 afforded 14 mg of compound 2 (Ambolic acid). The NMR data were recorded on Bruker Avance (400 MHz) NMR spectrometer in deuterated chloroform (CDCl_3_) with tetramethylsilane (TMS) as internal standard. The chemical structures of the compounds were deduced from ^1^H NMR and ^13^C NMR data and by comparison with the data reported for the compounds in previous studies.

### 4.3. HPLC-DAD Phenolic Profiling

The phenolic compounds in the plant extracts were detected and quantified using reversed-phase high-performance liquid chromatography (RP-HPLC) coupled with diode array detector (DAD). Known weights of each extract were dissolved in water:methanol (80:20) then filtered on sterile 0.20 μm disposable filter disk for liquid chromatography, and an Intertsil ODS-3 reverse-phase C18 column was used for the separation, employing a 1.0 mL/min solvent flow rate and 20 μL injection volume [73,74]. There were two mobile phases: A (0.5% acetic acid H_2_O) and B (0.5% acetic acid in CH_3_OH). A gradient elution was applied as follows: 0–10% B (0–0.01 min); 10–20% B (0.01–5 min); 20–30% B (5–15 min); 30–50% B (15–25 min); 50–65% B (25–30 min); 65–75% B (30–40 min); 75–90% B (40–50 min); 90–10% B (50–55 min). A photodiode array detector set at 280 nm wavelength was employed in the detection, and the UV data together with retention times were compared with authentic standards. Each analysis was performed three times. A calibration plot established through the elution of known concentrations (0.0, 0.00782, 0.01563, 0.03125, 0.0625, 0.125, 0.25, 0.5, and 1.0 ppm) of authentic compounds was used in the identification and quantification of the constituent phenolic compounds. A total of 26 phenolic standards (gallic, *p*-hydroxy benzoic, protocatechuic, ellagic, chlorogenic, *trans*-cinnamic, 3-hydroxy benzoic, vanillic, syringic, *p*-coumaric, rosmarinic, and ferulic acids; catechin, kaempferol, hesperetin, pyrocatechol vanillin, 6,7-dihydroxy coumarin, coumarin, rutin, myricetin, chrysin, luteolin, apigenin, taxifolin, and quercetin) were used. The results are expressed as μg per g dry weight of extract.

### 4.4. Antioxidant Activity

Four different methods, namely DPPH (2,2-diphenyl-1-picrylhydrazylhydrazyl) radical scavenging assay, ABTS (2,20-azino-bis-(3-ethylbenzothiazoline-6-sulfonic) acid) radical cation, CUPRAC (cupric reducing antioxidant capacity), and metal chelation assays, were used to measure the antioxidant potential of the plant extracts. Inhibition of lipid peroxidation was evaluated using the β-carotene-linoleic acid assay as described in a previous study [75]. Radical scavenging potentials on DPPH^•^ and ABTS^•+^ were measured by spectrophotometric means as previously described [57]. CUPRAC was determined in accordance with a method described elsewhere [76]. In the above assays, α-tocopherol and BHA (Butylated Hydroxyanisole) were employed as antioxidant standards against which the activities of the extracts were compared. EDTA was used as standard in the metal chelation assay performed on Fe^2+^ [77].

### 4.5. Cyclic Voltammetry Measurements

Cyclic voltammetry measurements were performed at room temperature using a Biologic SP-50 equipment (France) into an electrochemical cell using 5 μL of propolis in 20 mL ethanol [78]. A three-electrode system with a glassy carbon electrode (WE) of 3.0 mm diameter as a working electrode, Ag/AgCl_sat_ (3 M NaCl) electrode as a reference electrode (RE), and a platinum wire as a counter electrode (CE) was used. The potential applied was of E = ±1 V and, respectively, E = ±2 V vs. Ag/AgCl_sat_, with scan rate varied from 20 mV·s^−1^–100 mV·s^−1^. Before each run, the working electrode was polished with alumina paste down to 1 μm particle size on a polishing table and thoroughly cleaned with solvent. Three experiments for each condition were made to registered voltammograms.

### 4.6. Microbial Strains

The microorganisms used in this study are Staphylococcus aureus ATCC 25923, Enterococcus faecalis ATCC 29212, Listeria monocytogenes ATCC 7644, Pseudomonas aeruginosa ATCC 27853, Salmonella typhi ATCC 14028, Escherichia coli ATCC 25922, Candida albicans ATCC 10239, Candida tropicalis ATCC 13803, Chromobacterium violaceum CV12472 and Chromobacterium violaceum CV026, and Pseudomonas aeruginosa PA01.

### 4.7. Determination of Minimal Inhibitory Concentrations

MICs were determined by a microtiter broth dilution method as recommended by the Clinical and Laboratory Standards Institute [79]. The MIC was defined as the lowest extract or compound concentration that yielded no visible growth. The test medium was Mueller–Hinton broth, and the density of bacteria was 5 × 10^5^ colony-forming units (CFU)/mL. Cell suspensions (100 μL) were inoculated into the wells of 96-well microtiter plates in the presence of extract or compounds with different final concentrations (1, 0.5, 0.25, 0.125, 0.0625, 0.0312 mg/mL). The inoculated microplates were incubated at 37 °C for 24 h before being read.

### 4.8. Effect of Extract on Bacterial Biofilm Formation

The effect of the propolis extract and compounds at concentrations including 1, 1/2, 1/4, and 1/8 MIC on biofilm-forming ability of test microorganisms were tested with a microplate biofilm assay [80]. Briefly, 1% of overnight cultures of isolates were added into 200 μL of fresh Tryptose-Soy Broth (TSB) supplemented with 0.25% glucose and cultivated in the presence and absence of extracts without agitation for 48 h at 37 °C. The wells containing TSB+cells served as control. After incubation, the wells were washed with water to remove planktonic bacteria. The remaining bacteria were subsequently stained with 0.1% crystal violet solution for 10 min at room temperature. Wells were washed once again to remove the crystal violet solution. A volume of 200 μL of 33% glacial acetic acid or ethanol were poured in wells. After shaking and pipetting of wells, 125 μL of the solution from each well were transferred to a sterile tube, and the volume was adjusted to 1 mL with distilled water. Finally, optical density (*OD*) of each well was measured at 550 nm (Thermo Scientific Multiskan FC, Vantaa, Finland). Percentage of inhibition of the tested extracts was calculated using the formula:

$$\;\mathrm{Biofilm}\;\mathrm{inhibition}\;\left(\%\right)=\;\frac{OD550_{Control}\;-\;OD550_{Sample}}{OD550_{Control}}\times \;100$$

### 4.9. Bioassay for Quorum-Sensing Inhibition (QSI) Activity Using C. violacium CV026

Quorum sensing inhibition was evaluated as described elsewhere [81] with slight modifications. Five milliliters of warm molten Soft Top Agar (1.3 g agar, 2.0 g tryptone, 1.0 g sodium chloride, 200 mL deionized water) was seeded with 100 µL of an overnight CV026 culture, and 20 µL of 100 µg/mL C_6_HSL was added as exogenous AHL source. This was gently mixed and poured immediately over the surface of a solidified LBA plate as an overlay. Wells of 5 mm in diameter were made on each plate after the overlay had solidified. Each well was filled with 50 µL of sub-MIC concentrations of filter-sterilized extract or compounds. A white or cream-colored halo around this well against a purple lawn of activated CV026 bacteria was an indication of QSI. A clear halo indicated antimicrobial (AM) activity. The limit of detection of activity was also determined by applying serial dilutions of the extracts (1:1 to 1:8, using LB broth as diluent). End points were estimated as the lowest dilution of extract giving discernible inhibition of violacein synthesis. Each experiment was done in triplicate, and the assay plates were incubated at 30 °C for 3 days after which the diameters of the quorum sensing inhibition zones were measured.

### 4.10. Violacein Inhibition Assay Using C. violacium CV12472

Extracts of the propolis extract and compounds were subjected to qualitative analysis to find their QSI potentials against *C. violaceum* ATCC 12472 [9,57]. Overnight culture (10 µL) of *C. violaceum* (adjusted to 0.4 OD at 600 nm) was added into sterile microtiter plates containing 200 µL of LB broth and incubated in the presence and absence of sub-MICs of extract or compounds. LB broth containing *C. violaceum* ATCC 12472 was used as a positive control. These plates were incubated at 30 °C for 24 h and observed for the reduction in violacein pigment production. The absorbance was read at 585 nm. The percentage of violacein inhibition was calculated by the following formula:

$$\;\;\mathrm{Violacein}\;\mathrm{inhibition}\;\left(\%\right)=\frac{OD\;585\;control-OD585\;sample\;}{OD\;585\;control}\times 100$$

### 4.11. Swarming Motility Inhibition on P. aeruginosa PA01

Inhibition of swarming motility assay was done as described previously [82]. Briefly, overnight cultures of *P. aeruginosa* PA01 strain were point-inoculated at the center of swarming plates consisting of 1% peptone, 0.5% NaCl, 0.5% agar, and 0.5% of filter-sterilized D-glucose with various concentrations of extract or compound (50, 75, and 100 µg/mL), and the plate without the essential oil was maintained as control. Plates were incubated at an appropriate temperature in an upright position for 18 h. The swarming migration was recorded by following swarm fronts of the bacterial cells.

## 5. Conclusions

Infectious diseases remain one of the major causes of death in the worldwide, and this is becoming more complicated with notorious resistant strains. The emergence of bacterial resistance to antibiotics is a global health problem that has cause researchers to devise new strategies for developing new anti-infective drugs, capable of overcoming resistance and reducing bacterial virulence. Natural products and phytochemicals seem to be a remedy, and they are being investigated for their abilities to disrupt quorum sensing networks and bacterial biofilm formation. Propolis is a bioactive substance that is usually consumed as fractions or crude extracts due to its health benefits, and it is necessary to continuously report its chemical composition so to ensure quality control, safety, and therapeutic efficacy [83]. The antimicrobial activity of propolis on microorganisms, either gram-positive or gram-negative, has been extensively evaluated and reported with few papers on their effects on biofilms and quorum sensing. In this study, a propolis sample was evaluated for phenolic composition using HPLC-DAD, and the extract together with isolated cycloartane-type triterpenoid acids were evaluated for antimicrobial, antibiofilm, and anti-quorum sensing activities on pathogenic bacteria.

The samples showed variable antimicrobial effects on different microbial strains. Violacein inhibition on *C. violaceum* CV12472 were high while quorum sensing on CV026 were moderate, and propolis extract was more active than isolated compounds. The samples equally reduce swarming motility in flagellated *P. aeruginosa* PA01, indicating that they can reduce the incidence of surface colonization by bacteria. The results indicate that propolis can be a potential source of new era antibiotics that do not only inhibit bacterial growth but can also reduce the virulence factors and severity of infections.

## Figures and Tables

**Table 1:** Phenolic compounds detected in the propolis extract.

Compound Name	Linear Range (μg/mL)	LOQ (μg/mL)	µg/g Extract
**Quercetin**	6.25–400	10.35	43.53 ± 1.10
**Protocatechic acid**	1.56–100	12.07	40.76 ± 0.82
**Vanillic acid**	12.5–100	4.79	29.90 ± 1.05
**4-Hydroxybenzoic acid**	1.56–100	4.68	24.04 ± 0.21
**Luteolin**	3.00–30.0	3.99	4.44 ± 0.48
**6,7-dihydroxycoumarin**	3.13–200	7.56	TR
**Catechin**	30.0–120	19.33	TR
***p-*** **Coumaric acid**	1.56–100	3.06	TR
***trans*** **Cinnamic acid**	1.56–100	7.56	TR
**Vanillin**	12.5–100	8.13	TR

TR: traces; LOQ: Limit of Quantification in µg/mL.

**Table 2:** NMR data (ppm) of isolated compounds.

Position	27-Hydroxymangiferonic Acid (1)		Ambolic Acid (2)		Mangiferonic Acid (3)		Lupeol (4)		β-Amyrin (5)	
	^13^C	^1^H	^13^C	^1^H	^13^C	^1^H	^13^C	^1^H	^13^C	^1^H
**1**	33.6	1.85, 1.56	32.0	1.53, 1.22	33.4	1.85, 1.56	38.8	/	38.7	1.91
**2**	38.2	2.29, 2.75	30.4	1.76, 1.55	37.6	2.31, 2.71	27.5	/	23.6	/
**3**	218	/	77.9	3.30	214.8	/	79.0	3.19	79.01	3.24
**4**	55.0	/	40.5	/	50.3	/	39.9	/	37.2	/
**5**	42.8	2.15	47.1	1.29	48.4	1.69	55.3	/	55.3	/
**6**	21.2	1.56, 0.97	20.8	1.58, 0.79	21.5	1.53	19.3	/	18.0	/
**7**	28.2	1.90, 1.35	26.2	1.32, 1.08	25.7	1.34	34.2	/	32.8	1.57
**8**	47.8	1.61	47.9	1.51	47.9	1.50	41.1	/	41.5	/
**9**	21.0	/	19.7	/	21.1	/	50.5	/	47.6	/
**10**	25.7	/	26.0	/	25.9	/	37.2	/	36.8	/
**11**	26.7	2.25, 1.20	26.4	1.99, 1.12	26.7	/	21.2	1.40	28.1	1.94
**12**	32.8	1.65	32.8	1.60	32.8	1.64	25.3	1.88	121.8	5.18
**13**	45.4	/	45.3	/	45.4	/	38.5	/	145.3	/
**14**	48.8	/	48.6	/	48.8	/	42.8	/	42.1	/
**15**	35.5	1.29	35.5	1.30	35.5	1.35	27.2	/	26.6	/
**16**	25.5	1.35	27.9	1.86	28.2	-	35.6	/	31.1	/
**17**	52.2	1.61	52.5	1.61	52.2	1.61	43.0	/	40.8	/
**18**	18.2	0.99	18.0	0.98	18.1	1.08	48.3	/	50.5	1.94
**19**	29.7	0.61, 0.82	29.8	0.56, 0.34	29.6	0.58, 0.78	47.8	2.38	28.1	/
**20**	36.0	1.45	36.1	1.42	36.0	/	150.9	/	33.7	/
**21**	18.1	0.93	18.3	0.89	18.1	0.91	30.1	/	39.6	/
**22**	35.2	1.60	35.0	1.61	29.6	/	40.3	/	39.7	/
**23**	25.7	2.19	25.8	2.12, 1.93	34.8	/	28.4	1.04	28.1	0.80
**24**	130.0	7.02	148.9	/	145.8	6.9	15.6	0.97	16.7	0.91
**25**	149.2	/	45.6	3.18	126.6	/	16.1	1.40	15.6	0.77
**26**	171.2	/	178.9	/	173.0	/	16.0	0.84	16.8	0.94
**27**	57	4.37	16.3	1.31	11.9	1.85	14.5	0.79	23.2	1.15
**28**	22.2	/	24.9	0.97	22.2	1.04	18.1	1.26	17.5	1.08
**29**	20.8	1.08	14.1	0.81	20.8	1.10	109.3	4.56, 4.65	18.7	0.84
**30**	19.3	0.91	19.3	0.90	19.3	1.04	20.2	1.69	21.3	0.81
**31**	/	/	110.2	4.98, 4.92	/	/	/	/	/	/

**Table 3:** Antioxidant activity of propolis extract.

Test Sample	DPPH^•^ Assay	ABTS^•+^ Assay	CUPRAC Assay	Metal Chelating Assay
	IC_50_ (µg /mL)	IC_50_ (µg /mL)	A_0.5_ (µg/mL)	%Inh. (100 µg/mL)
*Extract*	14.90 ± 1.10	10.86 ± 0.91	6.50 ± 0.25	46.21 ± 0.71
***Standards***				
*α-Tocopherol*	12.26 ± 0.07	4.31 ± 0.10	10.20 ± 0.01	NT
*BHT*	25.37 ± 0.47	4.10 ± 0.06	3.80 ± 0.00	NT
*Quercetin*	2.07 ± 0.10	1.18 ± 0.03	NT	35.91 ± 0.82
*EDTA*	NT	NT	NT	85.40 ± 0.10

IC_50_ values for means ± SEM of three parallel measurements (*p* < 0.05). NT: not tested.

**Table 4:** Inhibition of violacein production in *C. violaceum* CV12472 of propolis extract and isolated compounds.

Sample	MIC (mg/mL)	Violacein İnhibition (%)					
		MIC	MIC/2	MIC/4	MIC/8	MIC/16	MIC/32
**Propolis extract**	0.5	100 ± 0.00	100 ± 0.00	85.26 ± 0.50	20.50 ± 0.10	-	-
**27-Hydroxymangiferonic acid**	1.0	100 ± 0.00	100 ± 0.00	100 ± 0.00	60.49 ± 0.25	35.89 ± 0.64	-
**Ambolic acid**	1.0	100 ± 0.00	100 ± 0.00	100 ± 0.00	45.22 ± 1.00	24.90 ± 1.00	-
**Mangiferonic acid**	0.5	100 ± 0.00	100 ± 0.00	82.60 ± 0.21	35.77 ± 0.31	-	-

-: no inhibition.

**Table 5:** Quorum sensing inhibition zones in *C. violaceum* CV026 of propolis extract and isolated compounds.

Sample	Anti-Quorum Sensing İnhibition Zones (mm)				
	MIC (mg/mL)	MIC	MIC/2	MIC/4	MIC/8
**Propolis extract**	0.25	18.0 ± 1.0	14.5 ± 0.5	-	-
**27-Hydroxymangiferonic acid**	0.5	12.0 ± 0.5	-	-	-
**Ambolic acid**	1.0	9.0 ± 1.0	-	-	-
**Mangiferonic acid**	0.5	12.3 ± 1.0	-	-	-

“-”: no inhibition.

**Table 6:** Swarming motility inhibition on *P. aeruginosa* PA01 by propolis extract and isolated compounds.

Sample	Swarming İnhibition (%)		
	100 µg/mL	75 µg/mL	50 µg/mL
Propolis extract	47.95 ± 1.11	25.65 ± 0.20	-
27-Hydroxymangiferonic acid	45.50 ± 1.20	16.62 ± 0.25	-
Ambolic acid	58.72 ± 1.05	31.80 ± 0.91	06.68 ± 0.70
Mangiferonic acid	40.78 ± 0.50	29.64 ± 0.25	08.47 ± 0.49

“-”: no inhibition.

**Table 7:** Antimicrobial activity (MIC values in mg/mL) of propolis extract and isolated compounds.

Sample	*S. aureus* ATCC 25923	*L. monocytogenes* ATCC 7644	*E. faecalis* ATCC 29212	*E. coli* ATCC 25922	*P. aeruginosa* ATCC 27853	*S. typhi* ATCC 14028	*C. albicans* ATCC 10239	*C. tropicalis* ATCC 13803
**PR**	0.25	>1	1	0.5	1	0.5	0.25	0.5
**1**	0.5	1	1	>1	>1	>1	0.5	1
**2**	0.5	>1	0.5	>1	>1	1	1	1
**3**	0.25	0.5	0.5	1	>1	1	0.5	0.25

**Table 8:** Antibiofilm activities of propolis extract and isolated compounds (% inhibition).

	PR				1				2				3	
	MIC	MIC/2	MIC	MIC/2	MIC/4	MIC/8	MIC	MIC/2	MIC/4	MIC/8	MIC	MIC/2	MIC/4	MIC/8
	Biofilm İnhibition (% İnhibition)													
***S. aureus***	18.3 ± 0.8	5.1± 0.2	40.5 ± 0.9	25.5 ± 0.2	8.8 ± 0.3	-	24.2 ± 0.3	5.5 ± 0.1	-	-	8.6 ± 0.2	-	-	-
***L. monocytogenes***	-	-	17.4 ± 0.1	5.5 ± 0.5	-	-	-	-	-	-	15.7 ± 0.2	-	-	-
***E. faecalis***	-	-	18.7 ± 0.4	5.4 ± 0.1	-	-	22.9 ± 1.0	-	-	-	32.4 ± 0.9	12.6 ± 0.2	-	-
***E. coli***	38.5 ± 1.2	20.5 ± 0.3	38.5 ± 1.1	25.8 ± 0.8	15.6 ± 0.6	8.3 ± 0.4	32.5 ± 0.5	21.8 ± 0.7	11.9 ± 0.2	3.3 ± 0.1	44.5 ± 1.0	28.9 ± 0.8	12.1 ± 0.2	5.2 ± 0.1
***P. aeruginosa***	20.6 ± 0.5	-	-	-	-	-	-	-	-	-	-	-	-	-
***S. typhi***	-	-	-	-	-	-	17.8 ± 0.3	-	-	-	14.2 ± 0.8	-	-	-
***C. albicans***	8.4 ± 0.3	-	13.5 ± 0.1	-	-	-	17.5 ± 0.6	-	-	-	44.5 ± 0.4	26.1 ± 0.5	-	-
***C. tropicalis***	10.9 ± 1.5	-	40.1 ± 1.0	21.8 ± 0.4	7.9 ± 0.1	-	39.8 ± 1.0	26.0 ± 0.9	10.2 ± 0.4	-	22.3 ± 0.8	5.8 ± 0.1	-	-

“-”: no inhibition. Test samples: Propolis extract (PR); 27-Hydroxymangiferonic acid (1); Ambolic acid (2); Mangiferonic acid (3).

## Data Availability

The data supporting reported results can be obtained from the corresponding author upon reasonable request.

## Sample Availability

Samples of the compounds **1**, **2** and **3** are not available from the authors.
